# Protonation Kinetics in Proteins at Basic pH Determined by pH-Dependent NMR Relaxation Reveal the Entire Relationship between Kinetics and p*K*_a_ Values

**DOI:** 10.1021/jacsau.5c00245

**Published:** 2025-05-13

**Authors:** Paula L. Jordan, Heiner N. Raum, Stefan Gröger, Ulrich Weininger

**Affiliations:** † Institute of Physics, Biophysics, 9176 Martin-Luther-University Halle-Wittenberg, D-06120 Halle (Saale), Germany; ‡ Department of Radiology, Medical Physics, University Medical Center Freiburg, Faculty of Medicine, University of Freiburg, D-79106 Freiburg, Germany; § Clinic for Radiology, University of Münster and University Hospital Münster, D-48149 Münster, Germany

**Keywords:** protonation, proton transfer, ionizable amino acids, NMR spectroscopy, NMR relaxation, lysines, tyrosines

## Abstract

Ionizable amino acid side chains in proteins undergo constant protonation and deprotonation reactions. These proton exchange dynamics are a fundamental feature of proteins and their electrostatic character, as well as the basis for many biological processes, such as general acid–base enzyme catalysis. Such dynamics have been measured in a site-specific way for aspartates, glutamates, and histidines by pH-dependent NMR relaxation experiments. Linear free-energy relationships between kinetic and thermodynamic parameters have been established that allow the description of proton-mediated proton exchange at low to neutral pH. Here, we complement the picture by determining the proton exchange kinetics of lysine and tyrosine side chains at basic pH. They display matching linear free-energy relationships that enable the description of hydroxide-mediated proton exchange at high pH. The underlying maximal second-order rate constants are approximately a factor of 40 higher for hydronium association compared to hydroxide dissociation. These combined findings provide a general framework for describing protonation kinetics, allowing for the prediction of protonation and deprotonation rate constants for ionizable groups with all possible p*K*_a_ values across the entire pH range.

## Graphical abstract

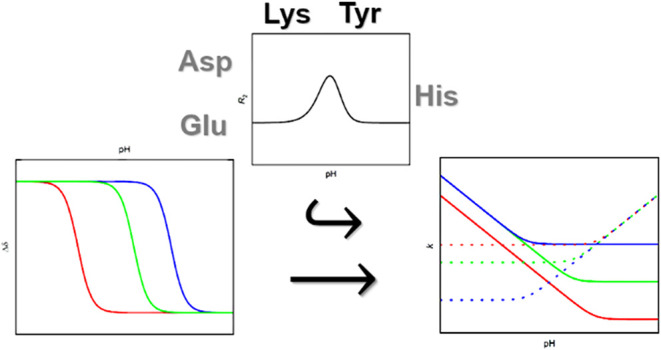

## Introduction

Proteins are dynamic entities whose properties and functions arise in large part from their noncovalent interactions.^1–3^ Electrostatic forces are particularly important due to their strength and long-range nature.^4,5^ There are seven amino acid side chains with ionizable groups between pH 0 and 14. Four (Asp, Glu, Tyr, and Cys) are predominantly negatively charged or neutral in solutions with pH values above or below their p*K*_a_ values, respectively. Three (His, Lys, and Arg) are predominantly positively charged or neutral in solutions with pH values below or above their p*K*_a_ values, respectively. Solution NMR spectroscopy has a long history in determining the protonation behavior (and the presence and absence of charges) of ionizable groups in a site-specific manner^6–9^ at equilibrium. Specifically, the chemical shifts of certain atoms in the ionizable amino acids depend strongly on the degree of protonation and thus are perfectly suitable probes for p*K*_a_ value determination.^10^ These experimental findings served as benchmarks for a large number of computational investigations of protein electrostatics.

In addition to the equilibrium of protonation and deprotonation of the ionizable groups (p*K*_a_ values), the kinetics between these states are of critical importance for the biophysical and functional properties of proteins. In fact, general acid–base catalysis and proton transfer are central to the vast majority of enzyme catalysis.^11,12^ For example, tyrosine plays a key role in many enzymes, like in aldose reductase,^13,14^ ketosteroid isomerase,^15,16^ or epoxide hydrolase,^17^ while lysine and arginine are important for DNA repair^18^ or cleavage.^19^ Proton exchange kinetics have been determined experimentally for individual side-chains by pH-dependent NMR relaxation for Asp and Glu at acidic pH in GB1 ^20^ and for His at moderately acidic to basic pH in a *ribbon-helix-helix* protein and T4-Lysozyme.^21^ The fundamental idea of proton exchange kinetics affecting relaxation or line shapes of certain amino-acid positions in a pH-dependent way, dates back to at least 1980.^22^ In addition, exchange kinetics at single pH values have been determined experimentally for His,^23^ Lys,^24,25^ and Arg^24^ or His coupled with conformational exchange.^26^

The small dimeric *ribbon-helix-helix* (*rhh*) protein from plasmid pRN1 of Sulfolobus islandicus consists of an β-strand formed by the two monomers followed by two α-helices per monomer.^27^ It displays an extremely high thermodynamic stability and is folded between pH 0 and 12.5.^9,28^ Unfolding is extremely slow and no alternative states have been experimentally detected, so no other processes interfere with the protonation kinetics.^9,28^ Thus, it is an ideal target for studying protonation kinetics at high pH, namely all eight Lys residues and two out of three Tyr residues. The p*K*_a_ values of the three Arg residues and the third Tyr are higher than pH 13 and thus, beyond the scope of the current study.

Here, we determine the proton exchange kinetics of Lys and Tyr side chains by measuring the pH dependence of transverse relaxation rate constants (*R*_2_) for distinct ^13^C resonances in each side chain. Our studies reveal the expected H_2_O/OH^–^ mediated exchange mechanism.^21^ Derived proton pseudo-first-order on-rate constants (*k*_on_) range from 0.4 to 4 × 10^6^ s^–1^, for p*K*_a_ values between 10 and 11, while second-order proton off-rate constants (*k*_off_) range from 0.2 to 5 × 10^10^ M^–1^ s^–1^. This closely agrees with the previously established linear free-energy relationship between kinetic (*k*_on_ and *k*_off_) and thermodynamic (p*K*_a_) parameters,^20,21^ and extends our understanding of proton exchange by the H_2_O/OH^–^ mediated exchange mechanism at high pH. Combining these findings with previously derived results^20,21^ yields to a complete and general picture of protonation kinetics over the entire pH range, based on aspartates, glutamates, histidines, and now lysines and tyrosines. This allows the calculation of explicit protonation rates at any pH value for all ionizable groups with all possible p*K*a values.

## Results and Discussion

The small dimeric *ribbon-helix-helix* protein contains eight Lys residues and three Tyr residues and is folded and stable up to pH 12.5.^9,28^ Their resonances have been assigned and the p*K*_a_ values of all ionizable residues have been determined previously^9^ using the macroscopic model,^29^ where multiple chemical shifts per residue were used to follow pH titrations. The p*K*_a_ values of all eight Lys residues and two out of three Tyr residues are amenable to investigation by pH titration between pH 0–12. The p*K*_a_ value of the third Tyr residue (Y47) was determined to be above pH 13 and thus serves as a negative control. Here, we monitor chemical shifts and transverse relaxation rate constants (*R*_2_) in response to pH perturbation for nuclei that exhibit a high chemical shift difference between protonated and deprotonated forms,^10^ namely Lys ^13^Cδ and Tyr ^13^Cζ (Figure S1). Using this approach, we are able to extract exchange terms (*R*_ex_) and consequently protonation exchange rate constants (*k*_ex_), as previously reported for Asp and Glu,^20^ as well as His^21^ residues. Since we are not interested in the interplay of intrinsic p*K*_a_ values as in the previous study,^9^ but rather in the actual point of 50% protonation, we fitted the p*K*_a_ values using the Hill model (Figures S2 and S3), exactly as in the approach for Asp and Glu.^20^ The Hill coefficient is cooperativity, describing the pH titration curve in addition to the p*K*_a_ value, which can be 1 (no change in cooperativity, no coupling between charged sides) and <1 (negative cooperativity, coupling between charged sides). Derived parameters are summarized in Table 1. pH titration curves for most residues were fit with Hill coefficients close to 1, except for K12 and Y16 with Hill coefficients around 0.8, and K32 with a Hill coefficient of around 1.5. Both K12 and Y16 are located on the same three-dimensional position of the intermolecular antiparallel β-strand, pointing in the same direction. Therefore, some negative cooperativity caused by Coulombic interaction between K12 and Y16 side chains would be expected. The positive cooperativity observed for K32, however, is likely an artifact of low data quality—primarily resulting from low cross-peak amplitudes combined with an incomplete transition between fully protonated and unprotonated states across the pH range used. Consequently, data for K32 were not analyzed further. However, the transverse relaxation rate could not be reliably analyzed anyway.

**Table 1:** Results of Chemical Shift Detected during pH Titration of Lys ^13^Cδ and Tyr ^13^Cζ

	p*K*_a_	n_H_	Δδ [ppm]
K6	10.79 ± 0.01	0.98 ± 0.02	4.63 ± 0.04
K12	10.54 ± 0.01	0.78 ± 0.01	4.68 ± 0.04
K30	10.86 ± 0.01	1.02 ± 0.01	4.96 ± 0.02
K32	10.48 ± 0.01	1.48 ± 0.06	3.66 ± 0.05
K45	11.27 ± 0.02	0.91 ± 0.01	6.91 ± 0.12
K53	10.25 ± 0.02	0.97 ± 0.03	5.73 ± 0.07
K55	11.08 ± 0.04	0.91 ± 0.04	5.46 ± 0.17
K56	10.96 ± 0.03	1.08 ± 0.05	4.71 ± 0.09
Y5	9.85 ± 0.01	0.99 ± 0.02	9.22 ± 0.09
Y16	9.85 ± 0.01	0.83 ± 0.02	10.22 ± 0.08
Y47	>13.7		

Studies on protonation exchange rate constants have shown, that H31 in T4-lysozyme, with a p*K*_a_ value of 9, undergoes protonation and deprotonation by a H_2_O/OH^–^ mediated (Figure 1, blue) exchange mechanism (with *k*_on_^H2O^ and *k*_off_^OH–^), in which H_2_O acts as the constant proton donor and OH^–^ as the pH dependent proton acceptor of the solvent.^21^ In contrast, residues with p*K*_a_ values of 7 and below undergo protonation and deprotonation by a H_3_O^+^/H_2_O mediated (Figure 1, red) exchange mechanism (with *k*_on_^H3O+^ and *k*_off_^H2O^).^20,21^ These two mechanisms can be distinguished by a shift of the maximum in *R*_2_ by 0.3 units toward higher pH than the p*K*_a_ value (H_3_O^+^/H_2_O mediated) or 0.3 units toward lower pH than the p*K*_a_ value (H_2_O/OH^–^ mediated).^20,21^

**Figure 1:**
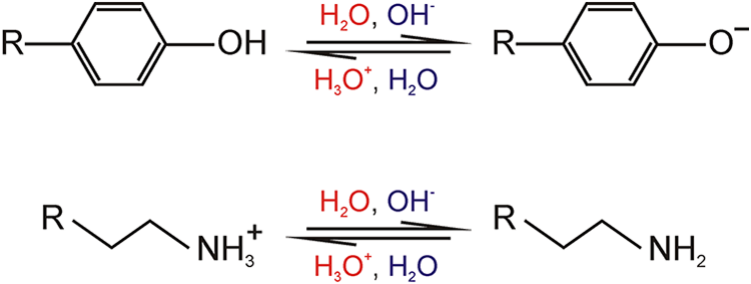
Proton exchange in tyrosine and lysine. The H_3_O^+^/H_2_O-mediated exchange mechanism is colored red, and the H_2_O/OH^–^-mediated exchange mechanism is colored blue.

### Protonation–Deprotonation Exchange in Lysines

Twenty-two *R*_2_ values for ^13^Cδ, as well as their corresponding chemical shift, have been acquired between pH 6 and 12. Their p*K*_a_ values and chemical shift differences (Δδ) were determined using the Hill model. Most titrations displayed a Hill coefficient close to 1. Furthermore, Hill coefficients can be disregarded in the *R*_2_ vs pH analysis, since they only affect the curves far from the p*K*_a_ value, where the transition is only visible by chemical shifts and not by *R*_2_, as has been shown previously.^20^ Three (K53, K55, and K56) of the eight *R*_2_ vs pH profiles displayed a combination of sufficient data quality and pronounced dispersion step (*R*_ex_) that they could be fit using the H_2_O/OH^–^-mediated exchange mechanism, eq 8 (Figure 2, red lines). For K53, and to a lesser extent K55 and K56, it can be clearly shown that the model without exchange (Figure S4) and the H_3_O^+^/H_2_O mediated exchange mechanism (Figure S5) do not describe the data. This agrees with the theoretical considerations of previous studies.^20,21^ Moreover, for three residues (K12, K30, and K45), it was possible to describe the data by manually choosing the minimal exchange rate (*k*_ex_) in agreement with the data. K6 and K32 could not be quantified in any meaningful way because of their insufficient data quality. The results of the fits and estimations are shown in Table 2. *k*_on_^H2O^ ranges from 0.4 to around 4 times 10^6^ s^–1^, while *k*_off_^OH–^ ranges from 0.03 to 0.5 times 10^10^ M^–1^ s^–1^, and there is a good agreement between fitted and estimated values.

**Figure 2:**
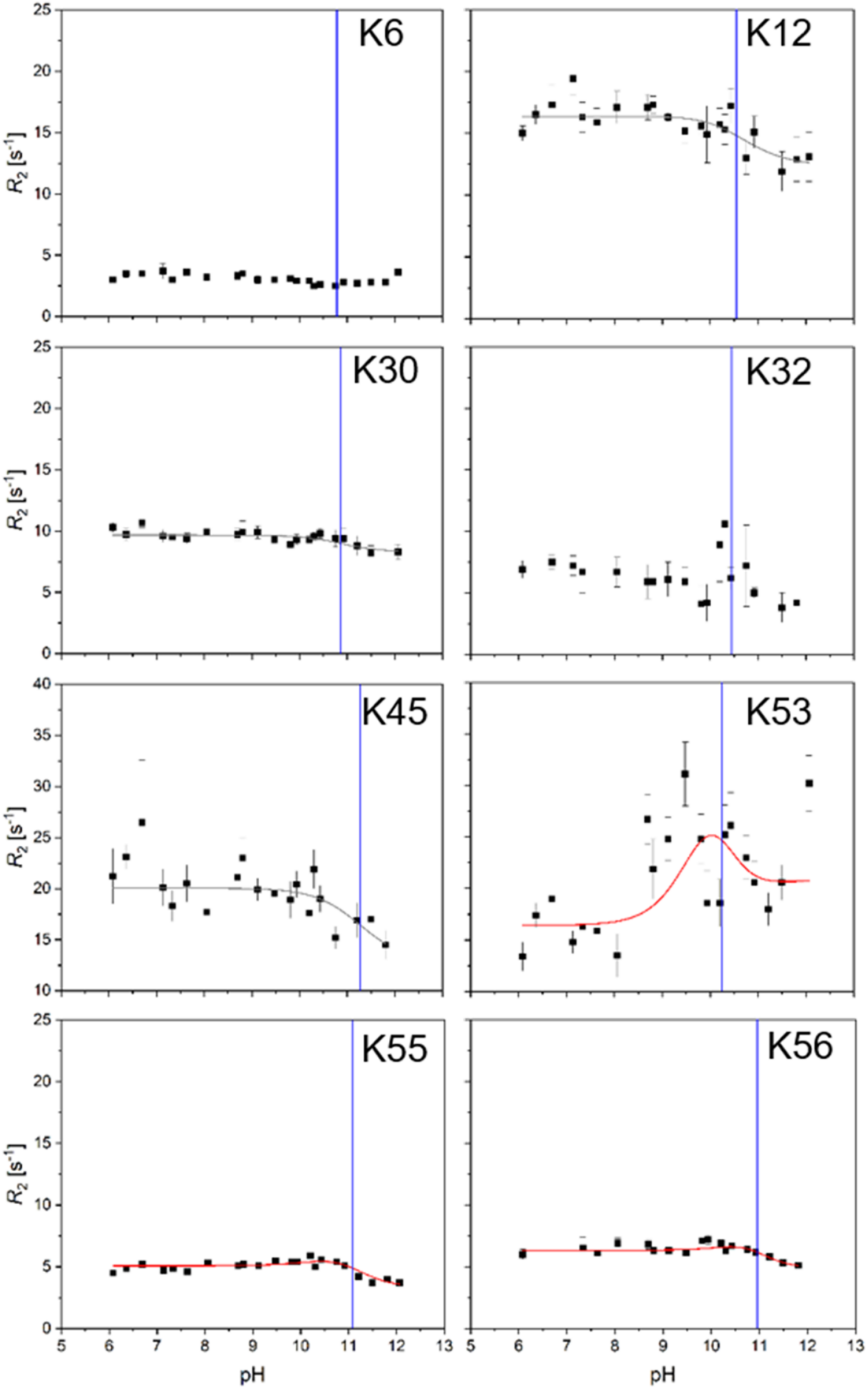
Proton exchange in lysine residues in *ribbon-helix-helix* protein. ^13^Cδ *R*_2_ vs pH profiles for all eight lysines are shown. Red lines (K53, K55, and K56) represent fits using the H_2_O/OH^–^-mediated exchange mechanism. Gray lines (K12, K30, and K45) estimates using the H_2_O/OH^–^-mediated exchange mechanism with minimal chosen *k*_ex_ values that agree with the data. Profiles for K6 and K32 could not be analyzed. p*K*_a_ values are shown as blue vertical lines. Results of the fitting and estimations are summarized in Table 2.

**Table 2:** *k*_on_ and *k*_off_ of Protons in Lys and Tyr Side-Chains

	*k*_on_^H2O^ [10^6^ s^–1^]	*k*_off_^OH–^ [10^10^ M^–1^ s^–1^]
K6	-	-
K12	>3	>0.4
K30	>4	>0.5
K32	-	-
K45	>2	>0.05
K53	0.43 ± 0.17	0.24 ± 0.09
K55	3.4 ± 0.7	0.28 ± 0.06
K56	3.1 ± 0.8	0.34 ± 0.08
Y5	2.0 ± 1.3	2.8 ± 1.8
Y16	3.5 ± 0.7	4.9 ± 1.0

### Protonation–Deprotonation Exchange in Tyrosines

Twenty-two *R*_2_ values for ^13^Cζ, as well as their corresponding chemical shift, have been acquired between pH 7 and 12. However, due to spectral overlap, only eight *R*_2_ values were sufficiently resolved for residue Y5. Two (Y5 and Y16) of the three *R*_2_ vs pH profiles displayed a combination of sufficient data quality and well-pronounced dispersion step (*R*_ex_) and could be fit using the H_2_O/OH^–^-mediated exchange mechanism, eq 8 (Figure 3, red lines). For Y16, it can be easily shown that the model without exchange (Figure S4) and the H_3_O^+^/H_2_O-mediated exchange mechanism (Figure S5) do not describe the data. Y47 with an estimated p*K*_a_ value above 13.7 does not show signs of titration up to pH 12 (Figure S3), and therefore could not be analyzed further. Because of lower data quality (fewer points with higher error) derived *k*_on_^H2O^ and *k*_off_^OH–^ values for Y5, with (2.0 ± 1.3) × 10^6^ s^–1^ and (2.8 ± 1.8) × 10^10^ M^–1^ s^–1^, respectively, should be treated with more caution, but generally agree well with *k*_on_^H2O^ and *k*_off_^OH–^ values for Y16, with (3.5 ± 0.7) × 10^6^ s^–1^ and (4.9 ± 1.0) × 10^10^ M^–1^ s^–1^. The results are shown in Table 2. Generally, *k*_on_^H2O^ values for both Tyr and Lys residues fall in the same range. However, *k*_off_^OH–^ are at least 10 times higher for Tyr compared to Lys residues, which explains the ∼1 unit lower p*K*_a_ values we observed for Tyr residues.

**Figure 3:**
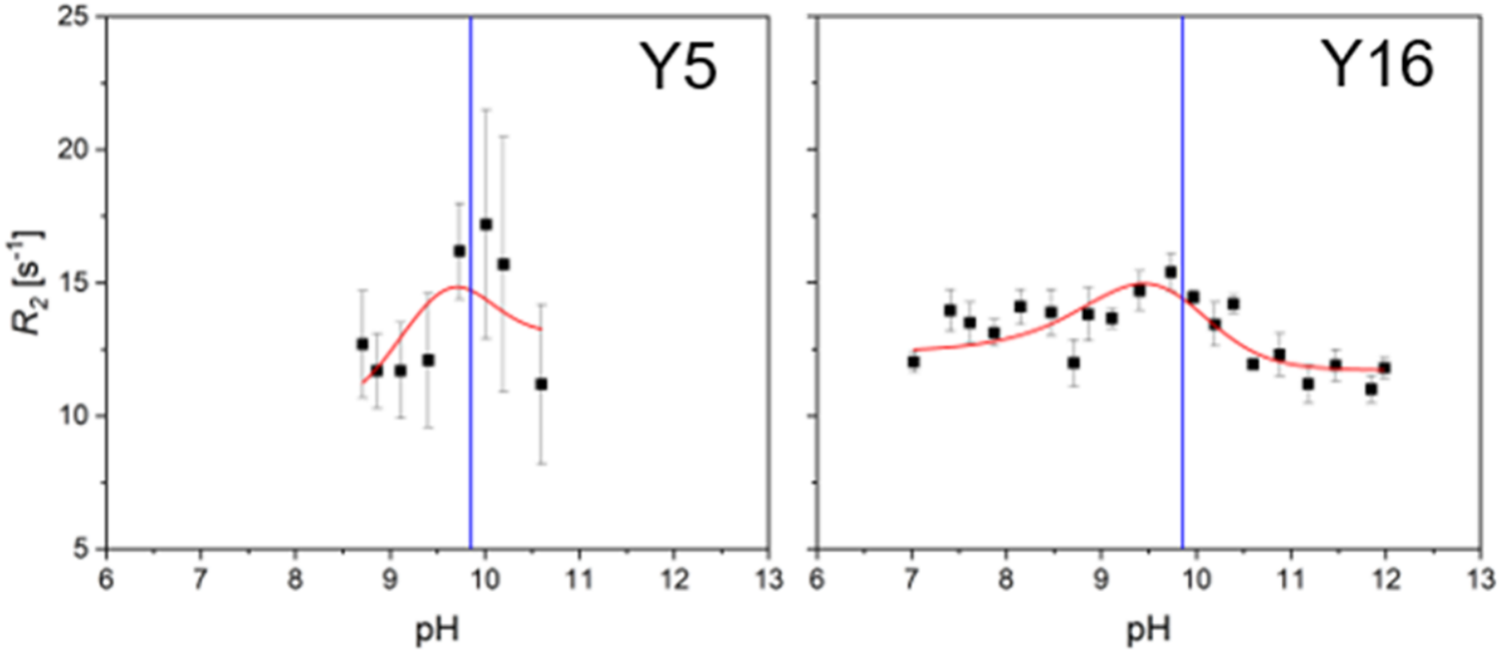
Proton exchange in tyrosine residues in *ribbon-helix-helix* protein. ^13^Cζ *R*_2_ vs pH profiles for two of the three tyrosines are shown. Red lines represent fits using the H_2_O/OH^–^-mediated exchange mechanism. p*K*_a_ values are shown as blue vertical lines. Results of the fitting are summarized in Table 2.

### Comparison of Exchange Rate Constants and p*K*_a_ Values: General Aspects and Linear Free-Energy Relationships

In order to put the obtained results in context and to draw more general conclusions, rate constants were plotted against their corresponding p*K*_a_ values (Figure 4), as has been done in previous studies on Asp, Glu,^20^ and His.^21^ This approach allows the comparison of kinetic and thermodynamic results using linear free-energy relationships. We also include data for Asp, Glu, and His residues from previous studies.^20,21^ Blue lines represent idealized slopes, in which only *k*_on_^H3O+^ (Figure 4A, up to p*K*_a_ 5) or *k*_off_^H2O^ (Figure 4B, starting from p*K*_a_ 5) change with their corresponding p*K*_a_ values, for the H_3_O^+^/H_2_O mediated proton exchange mechanism. This behavior has been observed previously,^21^ what remained unclear was the behavior for the H_2_O/OH^–^-mediated proton exchange mechanism, which will be addressed in this study. Here, *k*_off_^OH–^ (Figure 4A) for Lys, Tyr, and some His residues following the H_2_O/OH^–^-mediated proton exchange mechanism is described by a red line, where *k*_off_^OH–^ changes with p*K*_a_ above a p*K*_a_ of 10.4, while it remains unchanged below 10.4. Correspondingly *k*_on_^H2O^ (Figure 4B) can be described by a red line in which *k*_on_^H2O^ changes with p*K*_a_ below a p*K*_a_ of 10.4, but is unchanged above 10.4. In general, data for the H_3_O^+^/H_2_O and H_2_O/OH^–^ mediated proton exchange mechanism are mirror images, with higher kinetic rate constants (Figure 4B and Figure 4A, when multiplied by the H_3_O^+^ or OH^–^ concentration) at high or low pH. Proton exchange is slower around neutral pH. Second-order rate constants (Figure 4A) are about 40 times higher in the case of the H_3_O^+^/H_2_O-mediated proton exchange mechanism, indicating that it is more efficient than the H_2_O/OH^–^-mediated proton exchange mechanism. This is also reflected in the point of intersection of the pseudo-first-order rate constants at pH 7.8 (Figure 4B). Therefore, H_3_O^+^/H_2_O is the dominant exchange mechanism for pH 0–7.8, while the H_2_O/OH^–^ exchange mechanism is dominant for pH 7.8–14. Using the description of experimental data points by lines with idealized slopes, it is now possible to reasonably predict (depending on the accuracy of the fits in Figure 4) the kinetic rate constants of proton exchange from p*K*_a_ values. These predicted rate constants depend solely on the p*K*_a_ value and not on the type of ionizable group and further structural aspects of the protein. The chemical nature of the ionizable group and the three-dimensional structure of the protein determine the p*K*_a_ value, which in turn determines the kinetics. For ionizable groups not accessible to the solvent, there could potentially be lower kinetic rate constants. In such cases, the predicted rate constants represent estimates of the maximal possible exchange rate constants.

**Figure 4:**
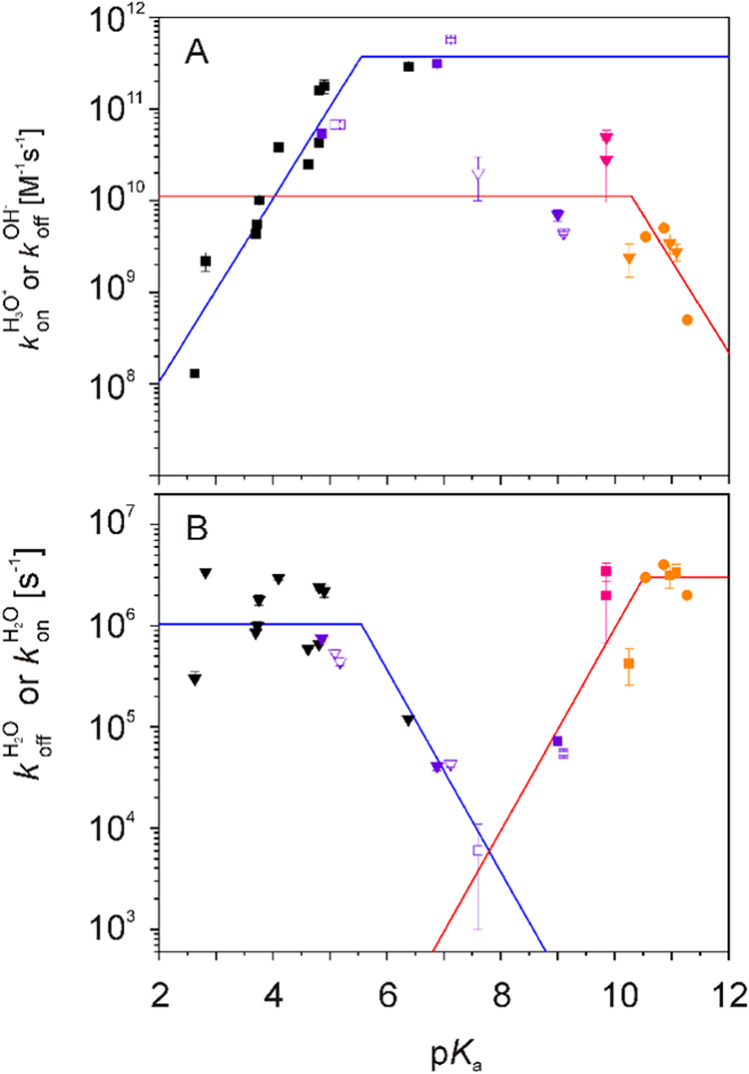
Proton exchange rate constants plotted vs p*K*_a_. Values for Asp and Glu^20^ are shown in black, for His in purple,^21^ for Tyr in pink, and for Lys in orange (also from left to right). Squares (A left, B right) and downward triangles (A right, B left) represent *k*_on_ and *k*_off_, respectively. Circles are the estimated values for Lys (these points are not included in the fits). Empty symbols for His represent values of the individual N positions in their side chains. (A) Second-order H^+^ on-rate constants (H_3_O^+^/H_2_O exchange mechanism) and H^+^ off-rate constants (H_2_O/OH^–^ exchange mechanism), both in units of M^–1^ s^–1^ (where the M unit refers to the concentration of H_3_O^+^ or HO^–^). (B) Pseudo-first-order H^+^ off-rate constants (H_3_O^+^/H_2_O exchange mechanism) and H^+^ on-rate constants (H_2_O/OH^–^ exchange mechanism) in units of s^–1^. Blue lines represent fits (to black and purple squares in A, and black and purple triangles in B) with ideal slopes (10^pH^, 0, −10^pH^), in which only the H^+^ on-rate or H^+^ off-rate changes with p*K*_a_, to values derived from the H_3_O^+^/H_2_O exchange mechanism.^20^ Red lines represent the fits (to purple, pink, and orange triangles in A, and purple, pink, and orange squares in B) for the H_2_O/OH^–^ exchange mechanism.

### Explicit Protonation Rates for Ionizable Groups with All Possible p*K*_a_ Values at Different pH Values

Having developed this general relationship between p*K*_a_ values and proton on- and off-rate constants allows the calculation of explicit protonation rates for all p*K*_a_ values over the entire pH range. Fitted proton on-rate constants are *k*_on_^H3O+^ = 10^p*K*_*a*_+6.02^ M^–1^ s^–1^ below p*K*_a_ 5.5 and *k*_on_^H3O+^ = 3.7 × 10^11^ M^–1^ s^–1^ above, and *k*_on_^H2O^ = 10^p*K*_*a*_–4.02^ s^–1^ below p*K*_a_ 10.4 and *k*_on_^H2O^ = 3.0 × 10^6^ s^–1^ above. Fitted proton off-rate constants are given as *k*_off_^OH–^ = 1.1 × 10^10^ M^–1^ s^–1^ below p*K*_a_ 10.4 and *k*_off_^OH–^ = 10^–p*K*_*a*_+20.3^ M^–1^ s^–1^ above, and *k*_off_^H2O^ = 1.0 × 10^6^ s^–1^ below p*K*_a_ 5.5 and *k*_off_^H2O^ = 10^–p*K*_*a*_ + 11.62^ s^–1^ above. An example calculation of p*K*_a_ values from 2 to 12 is shown in Figure 5. Proton on-rate constants range from 10^–2^ to 10^11^ s^–1^, while proton off-rate constants range from 10^0^ to 10^9^ s^–1^. At low pH, on-rates (mediated by H_3_O^+^) are the same for all p*K*_a_ values from 6–14, while the corresponding off-rate constants (mediated by H_2_O) are different. At high pH, the opposite behavior is observed. Off-rates (mediated by OH^–^) are the same for all p*K*_a_ values from 2–10, while the corresponding on-rate constants (mediated by H_2_O) are different. In other words, for H_3_O^+^ as the proton donor and OH^–^ as the proton acceptor, different p*K*_a_ values of the ionizable amino acid side chains do not affect proton transfer kinetics; the process is diffusion-limited. However, for H_2_O as the proton donor and acceptor, the different p*K*_a_ values of the ionizable amino acid side chains directly influence the proton transfer kinetics. Around neutral pH, where all four donor/acceptor mechanisms matter, the strongest effect of p*K*_a_ values on proton transfer kinetics is observed. However, the overall range of proton exchange rate constants at neutral pH is narrower, ranging from 10^1^ to 10^7^ s^–1^. In summary, Figure 5 provides a good estimate of the maximal proton on and off rate constants at any given pH, for any ionizable group with a known p*K*_a_ value. These rates can be compared to enzymatic rate constants and other proton-dependent processes.

**Figure 5:**
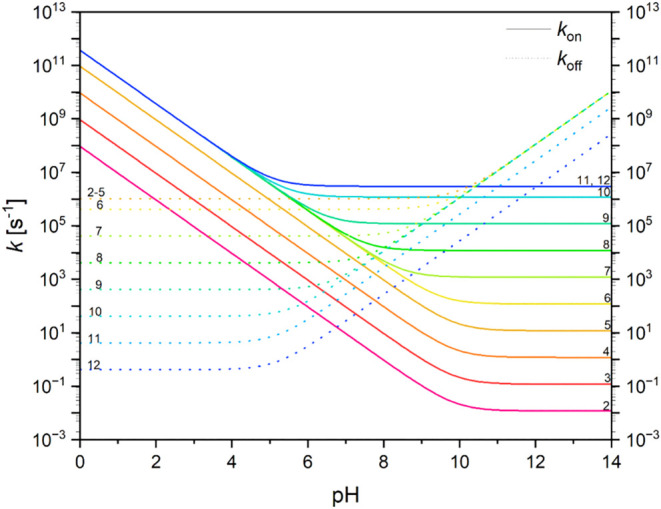
Proton exchange rate constants plotted vs pH for different p*K*_a_ values. Solid lines represent protonation and dashed lines represent deprotonation, based on experimentally found values. Different colors represent different p*K*_a_ values ranging from 2 (magenta) to 12 (dark blue).

## Conclusions

We developed site-specific ^13^C relaxation methods that allow to study proton exchange kinetics of Lys and Tyr residues. Protons in both ionizable side chains exchange by a H_2_O/OH^–^ mediated exchange mechanism, with proton on-rate constants between 0.4 and 4 × 10^6^ s^–1^ and proton off-rate constants between 0.2 and 5 × 10^10^ M^–1^ s^–1^. Kinetic (*k*_on_ and *k*_off_) and thermodynamic (p*K*_a_) parameters display a linear free-energy relationship that is complementary to the previously found relationship for the H_3_O^+^/H_2_O mediated proton exchange mechanism. Taken together, these findings provide a comprehensive quantitative framework (based on aspartates, glutamates, histidines, and now lysines and tyrosines) of proton exchange of all ionizable side chains in proteins across the entire pH range.

## Materials and Methods

### Protein Samples

The *rhh* protein from plasmid pRN1 of S. islandicus was expressed in M9 minimal media and purified as described previously,^9,30^ using 2 g/L ^13^C_6_ glucose for full ^13^C labeling of Tyr or 2 g/L unlabeled glucose and 1 g/L D-^13^C_4_ erythrose for site-selective ^13^C labeling of the δ position of Lys.^31^ Samples were concentrated to around 1.5 mM, the pH was adjusted directly on each sample in water without buffer (10% D_2_O) by HCl or NaOH and checked right before and after the measurement in the NMR tube and showed only minor deviations. No other components were present and no back-titrations were performed in order to keep the ionic strength as low as possible (<60 mM).^9^ pH values were measured at 25 °C using an inoLab pH 720 pH meter with a Hamilton Spintrode pH electrode calibrated with standard solutions of pH 4.006, 6.85, and 9.18.

### NMR Spectroscopy

All experiments were performed on a Bruker DRX 500 NMR spectrometer at a static magnetic field strength of 11.7 T and 25 °C. For measuring *R*_2_ of site-selective ^13^Cδ in lysines, a CH_2_ version of the HSQC-detected *R*_2_ experiment was acquired in an interleaved way (Figure S6). Intensity decays were sampled by 10 relaxation times: 0, 2, 4, 8, 16, 32, 64, 96, 128, and 200 ms. Each spectrum was recorded using 72 transients, 1258 Hz sampled by 196 complex points in ω_1_ (^13^C), 8012 Hz sampled by 1024 complex points in ω_2_ (^1^H), and a recycle delay of 1 s, resulting in a net acquisition time for each pH data set of 51.5 h. *R*_2_ of ^13^Cζ in tyrosines was measured by an H­(C)C type of HSQC detected *R*_2_ experiment, also recorded in an interleaved way (Figure S7). Intensity decays were sampled by 7 relaxation times: 0, 24, 48, 72, 96, 120, and 144 ms. Each spectrum was recorded using 96 transients, 5033 Hz sampled by 196 complex points in ω_1_ (^13^C), 11261 Hz sampled by 1800 complex points in ω_2_ (^1^H), and a recycle delay of 1 s, resulting in a net acquisition time for each pH data set of 50 h.

All spectra were processed with TopSpin and analyzed with PINT.^32^ Relaxation rates were derived by single exponential decays.

### Theory

Under the sample pH conditions used in this study, the dominant kinetic pathways for the protonation equilibria of lysine and tyrosine side chains can be described by^33^

1 $$\begin{array}{l} {\mathrm{RNH}}_{3}^{+}+{\mathrm{OH}}^{-}⇌{\mathrm{RNH}}_{2}+H_{2}O\\ \&\\ \mathrm{ROH}+{\mathrm{OH}}^{-}⇌{\mathrm{RO}}^{-}+H_{2}O \end{array}$$

The determined transverse relaxation rate is given as

2 $$R_{2}=p_{\mathrm{HA}}R_{2,\mathrm{HA}}+p_{A}R_{2,A}+R_{\mathrm{ex}}$$

where *p*_A_ and *p*_HA_ *=* 1 – *p*_A_ denote the relative populations of the deprotonated and protonated states. *R*_2,A_ and *R*_2,AH_ are the intrinsic transverse relaxation rates of the deprotonated and protonated states due to the chemical shift anisotropy and the dipole–dipole interaction. The contribution to the transverse relaxation rate due to chemical exchange is given by *R*_ex_. The exchange is to be expected in the fast exchange regime (*k*_ex_ > Δω), meaning *R*_ex_ is given by

3 $$R_{\mathrm{ex}}=\frac{p_{\mathrm{HA}}p_{A}{\left(\Delta \omega \right)}^{2}}{k_{\mathrm{ex}}}\left[1-2\frac{\tanh\left(\frac{k_{\mathrm{ex}}\tau_{\mathrm{CPMG}}}{2}\right)}{k_{\mathrm{ex}}\tau_{\mathrm{CPMG}}}\right]$$

with the delay τ_CPMG_ between the refocusing 180°-pulses in the CPMG block, the frequency difference Δω between the two states and the exchange rate *k*_ex_. The latter is given by^20,21^

4 $$k_{\mathrm{ex}}=k_{\mathrm{on}}^{H2O}+k_{\mathrm{off}}^{\mathrm{OH}-}\left[{\mathrm{OH}}^{-}\right]=k_{\mathrm{on}}^{H2O}\left(1+\frac{\left[{\mathrm{OH}}^{-}\right]}{K_{b}}\right)=\frac{k_{\mathrm{on}}^{H2O}}{p_{\mathrm{HA}}}$$

for H_2_O/OH^–^ mediated exchange mechanism with the on-rate constant *k*_on_^H2O^, the off-rate constant *k*_off_^OH–^ and the association constant

5 $$K_{b}=\frac{k_{\mathrm{on}}^{H2O}}{k_{\mathrm{off}}^{\mathrm{OH}-}}=\frac{p_{\mathrm{HA}}\left[{\mathrm{OH}}^{-}\right]}{p_{A}}$$

The relation between the acid–base dissociation constant *K*_a_ and *K*_b_ is given as

6 $$K_{b}=\frac{K_{a}}{[H^{+}]\left[{\mathrm{OH}}^{-}\right]}=\frac{[H^{+}]\left[{\mathrm{OH}}^{-}\right]}{K_{a}}$$

Under the given conditions (*k*_ex_ ≫ Δω and *k*_ex_ ≫ 1/τ_CPMG_) *R*_ex_ is well approximated by^20^

7 $$R_{\mathrm{ex}}=\frac{p_{A}p_{\mathrm{HA}}{\left(\Delta \omega \right)}^{2}}{k_{\mathrm{ex}}}=\frac{{\left(\Delta \omega \right)}^{2}}{k_{\mathrm{on}}^{H2O}}\frac{x}{{\left(1+x\right)}^{3}}$$

where *x* is given as *x = K*_b_[OH^–^]/*K*_b_. The function *R*_ex_(*x*) shows a local maximum for *x* = 0.5, which is equivalent to pH = p*K*_a_ *–* log_10_(2). Using the parameter *x*, eq 2 can now be rewritten as

8 $$R_{2}=R_{2,\mathrm{HA}}\frac{1}{1+x}+R_{2,A}\frac{x}{1+x}+\frac{{\left(\Delta \omega \right)}^{2}}{k_{\mathrm{on}}^{H2O}}\frac{x}{{\left(1+x\right)}^{3}}$$

The p*K*_a_ values as well as Δω were determined from the chemical shift titration curves

9 $$\delta_{\mathrm{obs}}=\frac{1}{1+x_{H}}\delta_{\mathrm{HA}}+\frac{x_{H}}{1+x_{H}}\delta_{A}$$

where δ_obs_ denotes the observed chemical shift and δ_HA_ and δ_A_ are the chemical shifts of the protonated and deprotonated state. The variable *x*_H_ is given as^20^

10 $$x_{H}={\left(\left[{\mathrm{OH}}^{-}\right]/K_{b}\right)}^{n_{H}}$$

with the phenomenological Hill coefficient *n*_H_. This definition takes the coupling between charged sides into account.

Besides the H_2_O/OH^–^-mediated exchange mechanism, there is also the H_3_O^+^/H_2_O mediated exchange mechanism

11 $$R_{2}=R_{2,A}\frac{1}{1+y}+R_{2,\mathrm{HA}}\frac{y}{1+y}+\frac{{\left(\Delta \omega \right)}^{2}}{k_{\mathrm{off}}^{H2O}}\frac{y}{{\left(1+y\right)}^{3}}$$

with *y* = [H^+^]/*K*_a_ was used to describe the measured data. The equation can be derived in the same manner as eq 8, but starting from the reaction

12 $$\begin{array}{l} R{\mathrm{NH}}_{3}^{+}⇌{\mathrm{RNH}}_{2}+H^{+}\\ \&\\ \mathrm{ROH}⇌{\mathrm{RO}}^{-}+H^{+} \end{array}$$

for the protonation equilibrium of the side chain amino group, where H^+^ denotes hydronium ions and higher-order complexes collectively.^18^ Here the corresponding rates for a H_3_O^+^/H_2_O mediated exchange mechanism are *k*_off_^H2O^ and *k*_on_^H3O+^.

### Data Analysis

pH profiles for chemical shifts and *R*_2_ were fitted with OriginLab 2019.

At each pH, *R*_2_ was determined by fitting a single-exponential function to the peak intensity decay for each side-chain. Error estimations were obtained as the standard deviation of the fit.

The parameters of eq 9 (δ_A_, δ_HA_, p*K*_a_, and *n*_H_) were fitted to the titration curves of the chemical shifts (δ_obs_ vs pH) using the Levenberg–Marquardt optimization routine implemented in OriginLab 2019. In the same way, the parameters of eq 8 (*R*_2,A_, *R*_2,HA_, and *k*_on_) were fitted to the experimental data sets of *R*_2_ vs pH under the assumption of [H^+^]­[OH^–^] = 10^–14^ M^–2^. Δω was kept fixed at the value obtained from the chemical shift titrations. Error estimates on the fitted parameters were obtained as the standard deviation of the fit to eqs 8 and 9, respectively.

## Supplementary Material



## Notes

Experimental pH titration curves for lysines and tyrosines; *R*_2_ vs pH profiles fitted by a H_2_O/OH^–^-mediated exchange without exchange; *R*_2_ vs pH profiles fitted by a H_3_O^+^/H_2_O-mediated exchange mechanism; pulse sequence for recording Lys side-chain ^13^Cδ *R*_2_ relaxation in selectively ^13^Cδ-labeled samples; pulse sequence for recording Tyr side-chain ^13^Cζ *R*_2_ relaxation in fully ^13^C-labeled proteins (PDF)
