# Supplementary material for: Protonation Kinetics in Proteins at Basic pH Determined by pH-Dependent NMR Relaxation Reveal the Entire Relationship between Kinetics and pK a Values
Source: JACS Au. 2025 May 13;5(5):2334–41. doi: 10.1021/jacsau.5c00245 (PMC12117459; doi:10.1021/jacsau.5c00245)
Supplement: Supplementary file 1 [file au5c00245_si_001.pdf]

Protonation Kinetics in Proteins at basic pH Determined by pH-Dependent NMR Relaxation Reveal the entire Relationship between Kinetics and pK_a values

Paula L. Jordan[1,2], Heiner N. Raum[1,3], Stefan Gröger[1], and Ulrich Weininger[1,*]

[1] Institute of Physics, Biophysics, Martin-Luther-University Halle-Wittenberg, D-06120 Halle (Saale), Germany

[2] Department of Radiology, Medical Physics, University Medical Center Freiburg, Faculty of Medicine, University of Freiburg, D-79106 Freiburg, Germany

[3] Clinic for Radiology, University of Münster and University Hospital Münster, D-48149 Münster, Germany

* Correspondence:

ulrich.weininger@physik.uni-halle.de

phone: +49 345 55 28555

fax: +49 345 55 27161

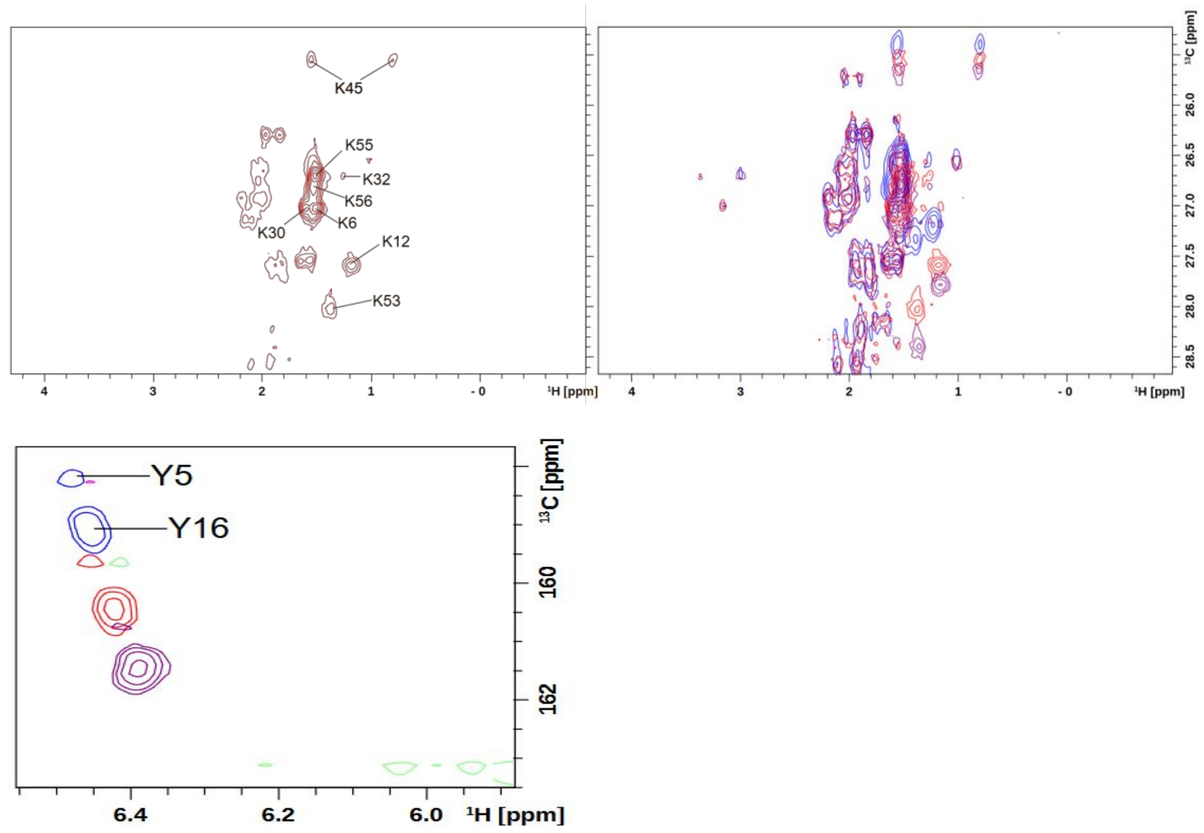

Figure S1. Exemplary spectra of the *rhh protein* at 25°C. Top: Lys $^{1}H\delta^{13}C\delta$ region of the aliphatic $^{1}H^{13}C$-HSQC. Top left: spectrum at pH 9.83 with labeled cross peaks of the eight Lys. Top right: spectra at pH 9.51 (blue), 9.93 (red) and 9.95 (purple). Bottom: Tyr region of Y5 and Y16 of the $^{1}H\varepsilon^{13}C\zeta$-HSQC spectra at pH 9.76 (blue), 10.04 (red) and 10.22 (purple).

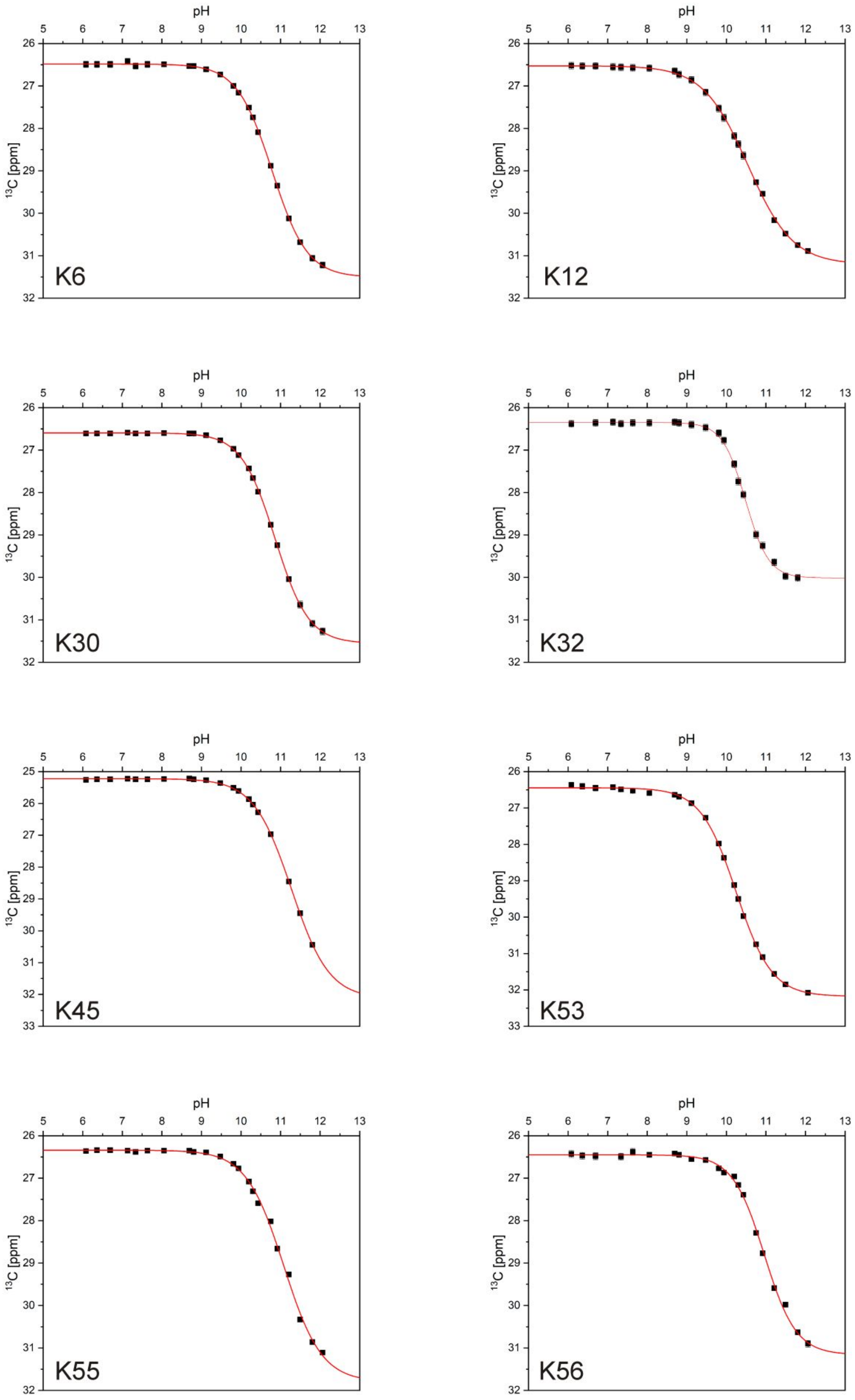

Figure S2. Experimental pH titration curves of the eight lysines monitored on ^{13}Cδ. Fits to $\delta_{\text{obs}} = \frac{1}{1+x_{\text{H}}}\delta_{\text{HA}} + \frac{x_{\text{H}}}{1+x_{\text{H}}}\delta_{\text{A}}$ with $x_{\text{H}} = ([\text{OH}^{-}]/K_{\text{b}})^{n_{\text{H}}}$ are shown as red solid lines.

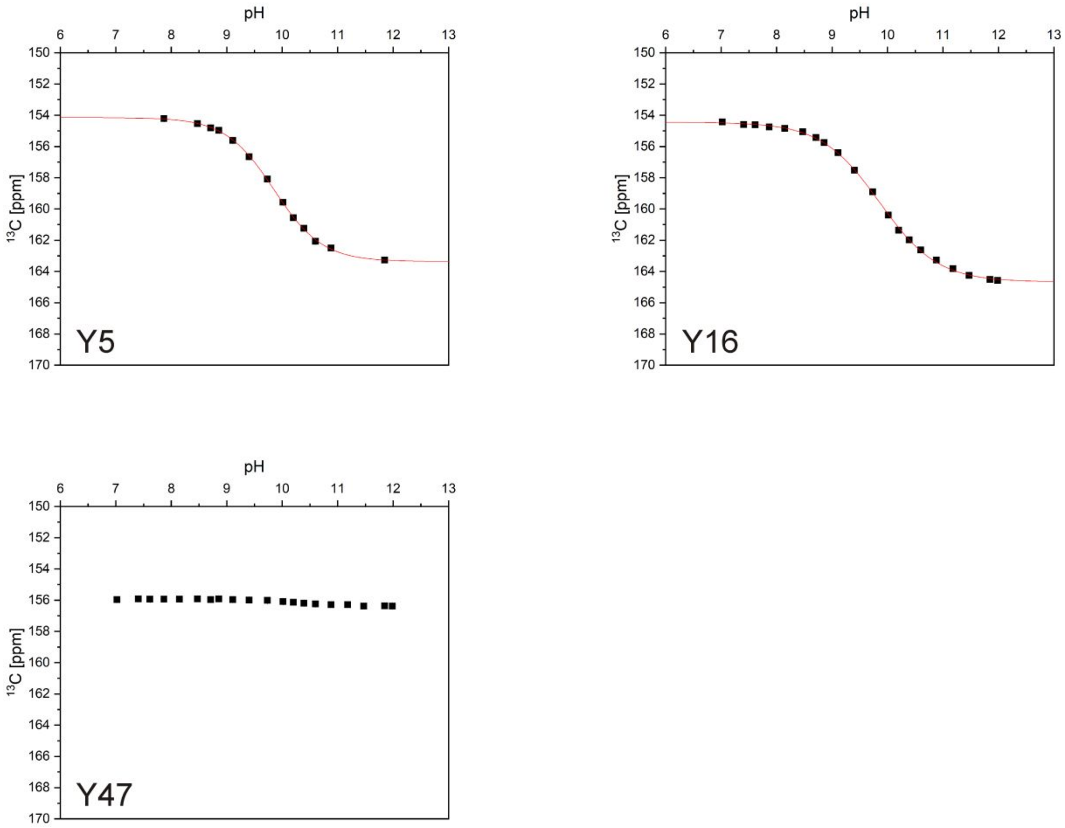

Figure S3. Experimental pH titration curves of the three tyrosines monitored on ^{13}Cζ. Fits to $\delta_{\mathrm{obs}} = \frac{1}{1+x_{\mathrm{H}}}\delta_{\mathrm{HA}} + \frac{x_{\mathrm{H}}}{1+x_{\mathrm{H}}}\delta_{\mathrm{A}}$ with $x_{\mathrm{H}} = ([\mathrm{OH}^-]/K_{\mathrm{b}})^{n_{\mathrm{H}}}$ are shown as red solid lines.

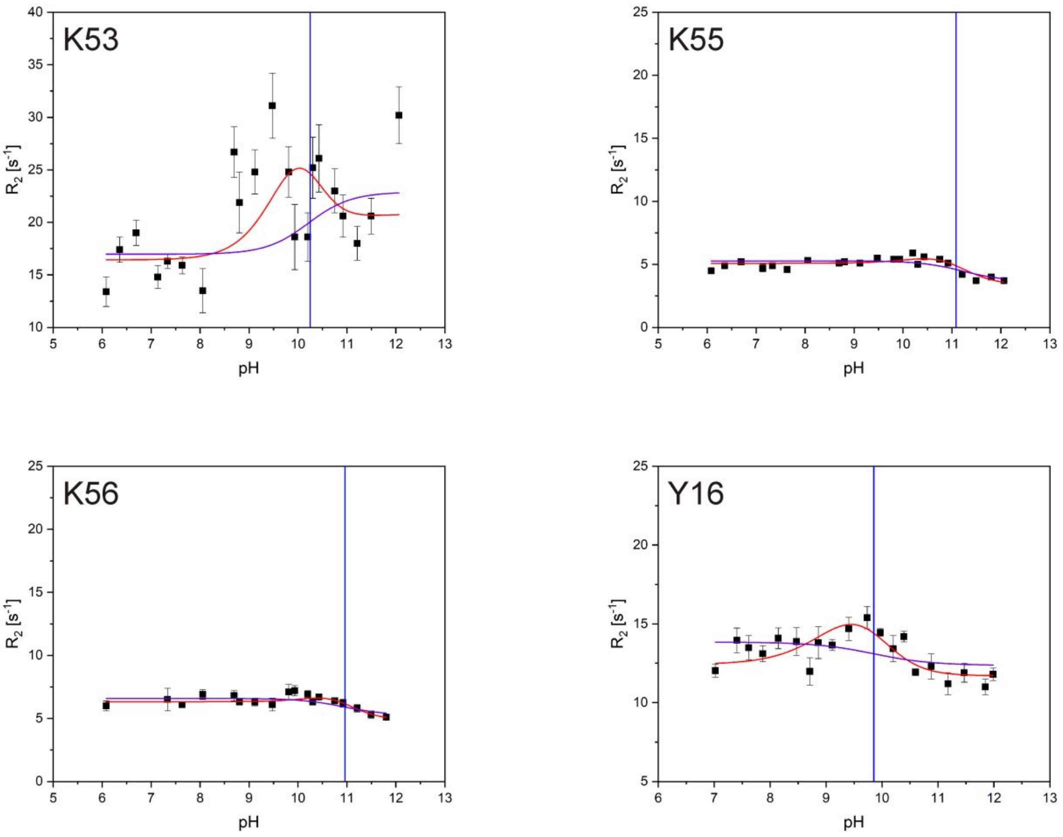

Figure S4. R_2 vs pH profiles fitted by a H_2O/OH^- mediated exchange mechanism (red) and without exchange (violet). Experimental pK_a values are shown as blue vertical lines.

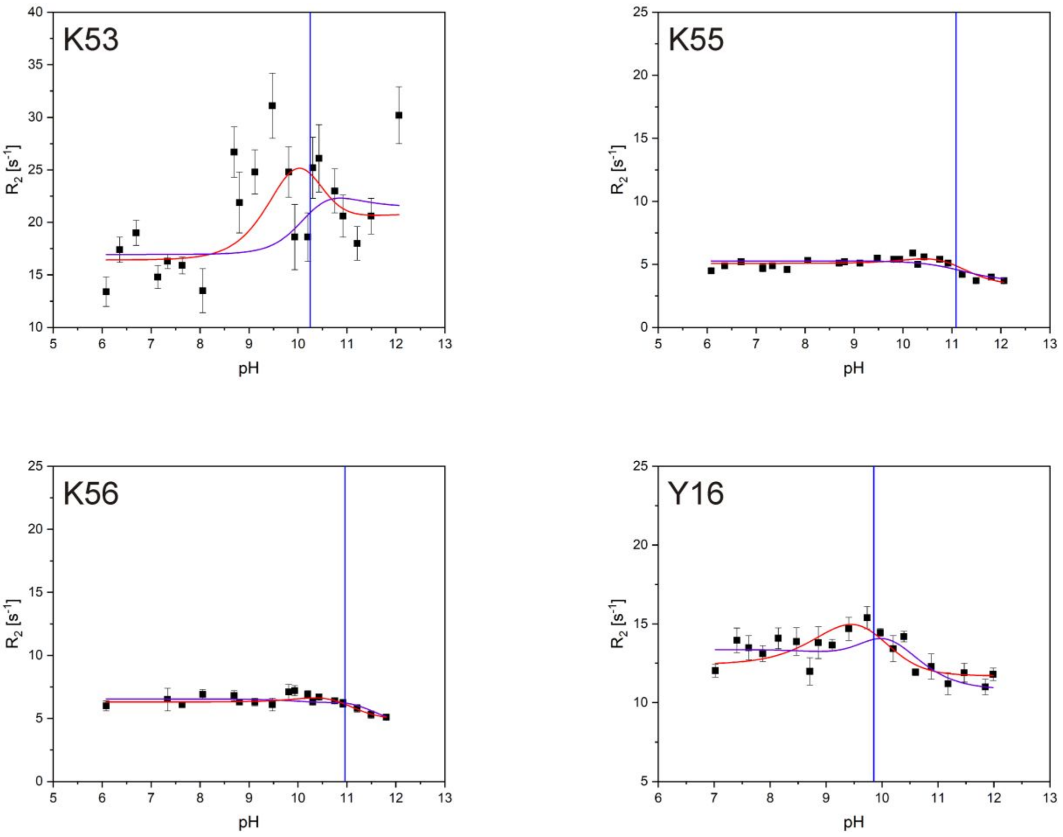

Figure S5. R_2 vs pH profiles fitted by a H_2O/OH^- mediated exchange mechanism (red) and a H_3O^+/H_2O mediated exchange mechanism (violet). Experimental pK_a values are shown as blue vertical lines.

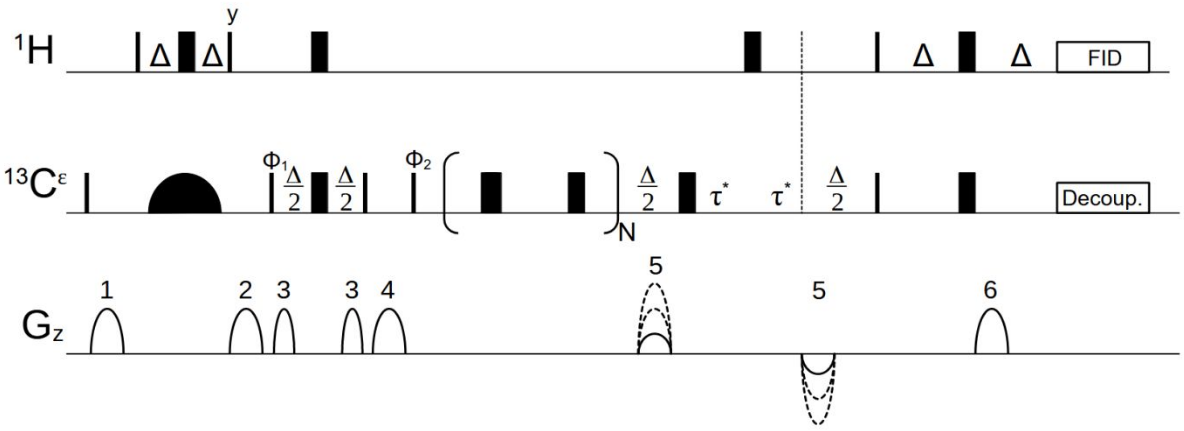

Figure S6. Pulse sequence for recording Lys side-chain ^{13}Cδ R_2 relaxation in selectively ^{13}Cδ-labeled samples. Narrow and wide vertical bars represent hard 90° and 180° pulses. The black half-ellipse denotes a REBURP centered at 29 ppm with a bandwidth of 13 ppm. The delay Δ has a length of 1850 µs. CPMG-pulses are applied every 500 µs. The delay τ^* is incremented to build up the indirect dimension. Gradient pulses are applied in z-direction, their durations (in ms) and relative power levels (in %) are set to (duration, power level) G1 = (0.8, 20), G2 = (0.8, 30), G3 = (0.5, 35), G4 = (0.8, -50), G6 = (0.8, -40.2), gradients used for the echo-antiecho-detection are shown as scattered lines G5 = (0.8, 80). Pulses are applied in x-direction unless indicated otherwise. The phase-cycles are given as Φ_1 = {x x -x -x}, Φ_2 = {y -y} and Φ_{rec} = {x -x -x x}. Decoupling during acquisition was achieved with GARP. Echo/anti-echo coherence selection was obtained by inverting the signs of gradient G5, every second increment Φ_2 and the receiver were inverted.

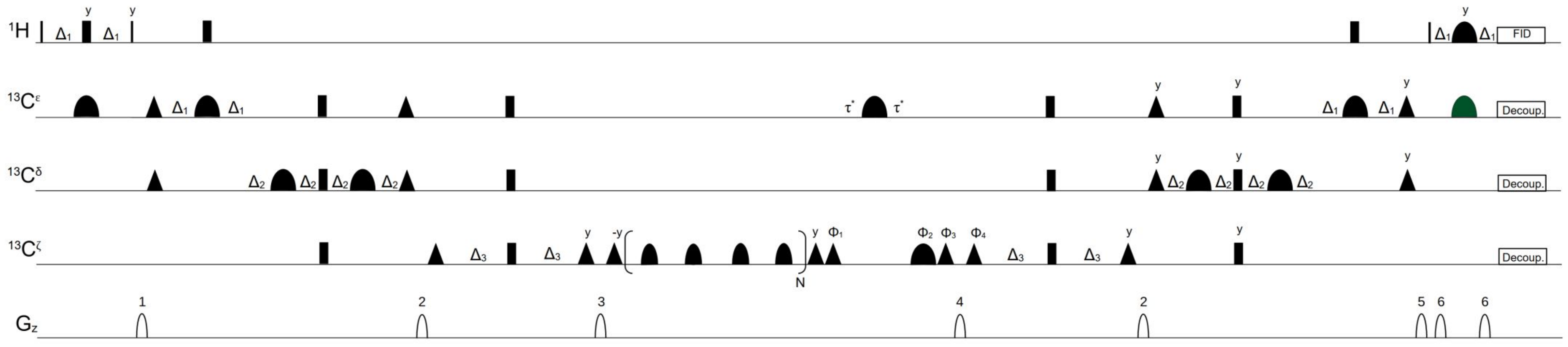

Figure S7. Pulse sequence for recording Tyr side-chain $^{13}C\zeta$ R_2 relaxation in fully ^{13}C-labeled proteins. Narrow and wide vertical bars represent hard 90° and 180° pulses. REBURP pulses applied on ^{13}C (black ellipses) have a bandwidth of 24 ppm and are centered at 115 ppm (Cε), 135 ppm (Cδ) or 165 ppm (Cζ) respectively. EBURP pulses (black triangles) have a bandwidth of 30 ppm and are centered at 115 ppm (Cε and Cδ simultaneously) or 165 ppm (Cζ) respectively. The last REBURP on $^{13}C\varepsilon$ (green ellipse) is centered at 115 ppm, has a duration of 1.3 ms and a bandwidth of 30 ppm. The REBURP on ^{1}H (black ellipse) is centered at 9 ppm with a duration of 1.5 ms and a bandwidth of 6.4 ppm. The delays have a length of Δ_1=1.39 ms, Δ_2=1.67 ms and Δ_3=4.46 ms. The distance between the 1.9 ms CPMG-pulses is 1.5 ms. The delay τ^* is incremented to build up the indirect dimension. Gradient pulses are applied in z-direction with a duration of 500 µs (narrow empty ellipses) and relative power levels (in %) of G1 = (11), G2 = (25), G3 = (40), G4 = (12), G5 = (15) and G6 = (60). Pulses are applied in x-direction unless indicated otherwise. The phase-cycles are given as Φ_1 = {x -x}, Φ_2 = {x x y y}, Φ_3 = {y}, Φ_4 = {x x x x -x -x -x -x} and Φ_{rec} = {x -x -x x -x x x -x}. Decoupling during acquisition was achieved with GARP. States TPPI coherence selection was obtained by incrementing Φ_1 and inverting the sign of Φ_3 and the receiver every second increment. We note that a 1.9 ms REBURP pulse is not an infinitely short pulse but provides accurate parameters in multiple CPMG relaxation dispersion experiments. In addition potential problems are not expected to matter under the fast exchange conditions of this study.
